# Supplementary material for: Increase in coercive measures in psychiatric hospitals in Germany during the COVID-19 pandemic
Source: PLoS One. 2022 Aug 31;17(8):e0264046. doi: 10.1371/journal.pone.0264046 (PMC9432719; doi:10.1371/journal.pone.0264046)
Supplement: Attachment — Submitted filename: Response to reviewers.docx [file pone.0264046.s001.docx]

Response to reviewers

*Dear editor,*

*we thank you and the reviewers for the careful reviewing of our manuscript and the suggestions that were very helpful for us to improve the clarity and comprehensibility of our manuscript. We answer point by point and show changes in our manuscript highlighted in yellow, accompanied by the tracked version of the revision:*

1. We ensured that our manuscript meets PLOS ONE’s style requirements.

- Titles and abbreviations were removed from the authors and from the affiliations.
- Physical address was removed from the Corresponding author, initials were added behind the email address.
- Therefore, we changed our Level 1 (2) Headings to 18 (16) pt font and removed the numbering. Italics are not used anymore.
- Empty rows in tables were removed. A standard font with standard font size (Arial, 11 pt) is now used in the tables.
- References are written in brackets. References with more than six authors list the first six authors followed by “et al.”

2. In your Data Availability statement, you have not specified where the minimal data set underlying the results described in your manuscript can be found. PLOS defines a study's minimal data set as the underlying data used to reach the conclusions drawn in the manuscript and any additional data required to replicate the reported study findings in their entirety. All PLOS journals require that the minimal data set be made fully available. For more information about our data policy, please see <http://journals.plos.org/plosone/s/data-availability>.

*We have created the deposit, where the data will be checked as soon as the ms will be accepted. It is provisionally accessible under the following link:*

<https://datadryad.org/stash/share/br-EupCXbygnoCUkVyfLKSDVgqOL86nBxDjgYbqIdF4>

Upon re-submitting your revised manuscript, please upload your study’s minimal underlying data set as either Supporting Information files or to a stable, public repository and include the relevant URLs, DOIs, or accession numbers within your revised cover letter. For a list of acceptable repositories, please see <http://journals.plos.org/plosone/s/data-availability#loc-recommended-repositories>. Any potentially identifying patient information must be fully anonymized.

Important: If there are ethical or legal restrictions to sharing your data publicly, please explain these restrictions in detail. Please see our guidelines for more information on what we consider unacceptable restrictions to publicly sharing data: <http://journals.plos.org/plosone/s/data-availability#loc-unacceptable-data-access-restrictions>. Note that it is not acceptable for the authors to be the sole named individuals responsible for ensuring data access.

We will update your Data Availability statement to reflect the information you provide in your cover letter.

3. We note that you have stated that you will provide repository information for your data at acceptance. Should your manuscript be accepted for publication, we will hold it until you provide the relevant accession numbers or DOIs necessary to access your data. If you wish to make changes to your Data Availability statement, please describe these changes in your cover letter and we will update your Data Availability statement to reflect the information you provide.

Additional Editor Comments (if provided):

Dear Author, Study entitled-Increase in coercive measures in psychiatric hospitals in Germany during the COVID19 Pandemic by Tilman Steinert et al 2022. Study examine changes in psychiatric hospital cases with effect of pandemic regarding the percentage of patients exposed to coercive interventions, and aggressive incidents. Study shows that increased percentage of patients exposed to coercion is not only due to a decreased percentage of voluntary patients, as the duration of coercive measures per case also increased. The focus of attention has shifted from prevention of coercion to prevention of infection.
Article is excellent for the psychiatric perspective, presented in an intelligible fashion and written in standard english however, technical and statistical explanation required extensively for the better understanding of the article. I had appended the reviewer comment for the better understanding and to make article in better shape.
Along with the reviewer comment following suggestions are also given below for the article.
1. Understanding working culture of people, varied clinical complications and regional risk is important for the study (Mental effect of covid-19 pandemic on affected, non affected and families of the affected. Gupta, A 2020. International Journal of Research in Pharmaceutical Sciences. 11(Special Issue 1), pp. 1804-1808.
2. Study of the psychiatric through behavioral changes is also important to understand the risks (COVID-19: Risk of alcohol abuse and psychiatric disorders. Haddadi, S., Murthi, M., Salloum, I., Mirsaeidi, M.S. 2020. Respiratory Medicine Case Reports. 31,101222.
3. Advanced treatment pattern, suggestions, technologies and future prospects of the study will add on more to make the article in better shape.
(Telephone information service for psychiatric patients during the covid-19 pandemic: Experience with a direct phone line in the nyírő gyula national institute of psychiatry and addictions in hungary | [Beteginformációs telefonvonal a covid-19-járvány idején: A nyírő gyula-opai-ban működtetett közvetlen vonallal szerzett tapasztalataink]. Katalin, C., Viktor, B., Krisztina, L., (...), Tünde, V., Szabolcs, K. 2020. Neuropsychopharmacologia Hungarica. 22(4), pp. 166-171. And Virtual reality exposure therapy (Vret) for anxiety due to fear of covid-19 infection: A case series. Zhang, W., Paudel, D., Shi, R., (...), Zhou, Y., Zhang, B. 2020. Neuropsychiatric Disease and Treatment. 16, pp. 2669-2675).

*Dear editor,*

*We understood the necessity for further clarifications. We expressed now more clearly that COVID 10 had little direct impact of hospital admissions, because COVID-related depressive and anxiety disorders are typically referred to outpatient treatment and do not require hospitalisation. On the other hand, video techniques etc. are used for out-patients, not for in-patients. Our study question is related to in-patients only.*

1. *We tried to go into detail as regards Covid-19 as a risk factor for mental health conditions (we have taken the liberty of citing references dealing with high income countries which are more comparable to the situation in Baden-Wuerttemberg/Germany).*
2. *… as well as mental health conditions as a risk factor for a severe course of an infection with Corona-SARS2-virus.*
3. *We also looked at the impact on treatment and support services for the chronically mentally ill.*

*We inserted the following sentences into the introduction section:*

In Germany, psychiatric clinics are widely available on a high quality level. About half of these clinics are part of university hospitals or general hospitals, the other half are specialized psychiatric hospitals. Day clinics are widely available, standing alone or as a part of these hospitals. Outpatient treatment is provided by the hospitals for people with severe mental disorders together with community psychiatric services, treatment for milder mental disorders is provided by physicians or psychologists in own practice, funded by health insurances. Community treatment orders or other types of involuntary outpatient commitment are not legalized, so that any type of coercion in association with treatment of mental disorders can only occur within a hospital. […]

Surprisingly, mental health in large segments of society did not appear to have deteriorated significantly as a result of the pandemic. In particular, people who were already unwell before the pandemic did not see them deteriorate further in studies. On the other hand, there continue to be warning voices that see a psychiatric pandemic heading toward society.

*Further, we inserted the following sentences into the discussion section:*

Notably, cases of severe mental disorders directly caused by an infection with COVID-19 or anxiety associated with the pandemic, vaccinations or lockdown measures were anecdotally reported (21) but had little impact on the number of in-patient admissions. The frequently observed depressive and anxiety disorders were mostly treated in out-patient services, also by video consultations (22) and in newly established services for this purpose in Baden-Wuerttemberg (23).

*Please accept that we referred mostly to references from Germany in this context, (23) was an intervention that was conducted exactly in the same regions (but referred to out-patients)*

Decision- Major revision with response to all reviewers comment pointwise.


Reviewers' comments:

Reviewer's Responses to Questions

**Comments to the Author**

Review Comments to the Author

Reviewer #1: Although Work is good and well But there are some points, after improvment of those paper can be acceptable.

1.Explain the method of sample size determination

*This was not a sample, but a full survey of a 11 million-State over 24 months. Maybe we had created confusion, naming 32 and 31 hospitals, respectively, in the text. One hospital was not included because it is licensed only for children and adolescents and our study question addressed treatment of adults. We tried to clarify this by extending the section study design as follows:*

We used an observational prospective design and compared data of Baden-Wuerttemberg’s 31 licensed hospitals treating adults (one university hospital is only licensed for child- and adolescent psychiatry and therefore was excluded from this analysis) on coercive measures, forced medication, and aggressive incidents in adult psychiatry from 2019 (before the pandemic) with data from the first year of the pandemic (2020). Forensic psychiatry is also part of the registry, but was not included in this analysis. Hence, this is a full survey, comprising each coercive measure that had been performed in one of the State’s psychiatric hospitals in the respective year in adults. Baden-Wuerttemberg with 11 million inhabitants covers the South West of Germany, with Stuttgart as the State’s capital. Data are available for the respective years, but, due to data privacy rules, the exact date of incidents is not provided so that we could not restrict our analysis to the months of the pandemic (beginning in March, 2020). This may have led to a systematic underestimation of observed changes of about 15%.

2. Define clearly different type of samples.

*As indicated, the two data sets compared are not samples but complete data sets each for one calendar year (2019/2020).*

3. Result and statistical analysis is not properly clear.
*We followed the detailed suggestions as provided by reviewer #2 and #3 for clarifications.*

4. Discussion is not clearly written

*Again, we followed the detailed suggestions as provided by reviewer #2 and #3 for clarifications.*

Reviewer #2: Authors look at the topic of increases in coercive measures during COVID-19 and find use of some practices increased while others decreased as a trade-off in treatment quality. The contrast in practices was interesting to read in the abstract. Possible reasons for this change could be expanded upon in some way.

Authors provide research from their work showing trends have decreased slightly over time, so this seems like an important follow-up study to consider the impact of the pandemic on rates. While there isn’t much literature on rates that has been published, it may help if authors could discuss a bit more broadly on the impact of the pandemic on care quality to help frame or present a stronger rationale for the expected change in treatment quality rates.

*Treatment quality seemed to be altered in different parts of the health systems affecting different diagnosis groups (e. g. Parkinson’s disease, chronic pain, ischemic strokes) and different outcomes (e. g. treatment delays).*

*We added the following paragraph to the discussion:*

A similar development was observed in most medical specialties [9,10] where it was also followed by a decline of treatment quality, especially a delay of treatment as well as a higher burden of disease.

Authors discuss important points and limitations in using the data that make it hard to do research in this area given reporting practices which is helpful to address upfront. I’ve had similar issues with getting “real” values at the patient or incident level as well (and around the right time frame), so appreciate the description.
Authors mention the SOAS-R measure in the Methods. It was unclear if or how this fit within the context of the study. Up to that point, the focus seemed to be on coercive measures (although now re-reading the abstract it is a bit clearer). Further background and description of this measure and its consideration in the study would be helpful
if retaining in the manuscript.

*We extended the paragraph on the SOAS-R as follows:*

The Staff Observation Aggression Scale – Revised (SOAS-R) was introduced for regular use and reporting in 10 out of the 31 hospitals within the last decade. The SOAS-R is a one-page form that can be filled within a few minutes by staff without training after an aggressive incident. It comprises five domains: provocation (score 0-2), means used by the patient (score 0-3), target of aggression (score 0-4), consequence(s) for victim(s) (score 0-9), and measure(s) to stop aggression (score 0-4). The possible range of the total score results in 1-22 points. The scale has been extensively used in research on violent incidents in psychiatric hospitals in Europe in the past 30 years. Characteristics of the scale and methods of recording have been reported in detail elsewhere (13-15). Due to some doubts with respect to fully covering self-directed aggression, we restrict the analysis to aggression toward others and toward objects as indicated in domain 2, target of aggression.

In the study design section, authors should note having data on SOAS-R from 10 hospitals (mentioned earlier in the methods).

*We removed that from the paragraph on the SOAS-R and added the following sentences to the study design:*

In addition, we could collect aggressive incidents as recorded with the SOAS-R from 10 of the 31 hospitals. These ten hospitals belong to a State-run company. Most of them are divided into several sites, and together they serve about half of the 11 million population. The data structure and ways of data collecting were very similar as described for coercive measures. However, the dataset does not allow for relating reports on aggressive incidents and coercive measures on individual patient level.

Analysis plan seems fairly straight forward. It would help if some additional considerations were part of this approach. It seemed like authors had collected more data about the characteristics of patients at each site. It would help to discuss whether patterns were observed consistently across hospitals or if some places saw different patterns than others.

*Dear Reviewer, thank you. There is another very recent paper of ours dealing with patterns across hospitals (Flammer E, Hirsch S, Thilo N, Steinert T. “Our Patients Are Different”: Predictors of Seclusion and Restraint in 31 Psychiatric Hospitals. Front. Psychiatry, 26 April 2022 | https://doi.org/10.3389/fpsyt.2022.791333). We found the percentage of involuntary admissions to be the only significant variable in the multivariable model for prediction of patients subjected to coercion. The largest proportion of the heterogeneity of coercion was unexplained in our models so we can’t draw further conclusions on that.*

*We also added further information on variation between hospitals:*

The percentage of involuntary cases varied between hospitals from 0.2% to 26.0% in 2019 and from 0.3% to 34.3% in 2020. The percentage of cases subjected to any kind of coercive measure varied between hospitals from 0.1% to 10.3% in 2019 and from 0.2% to 12.7% in 2020.

*And, regarding aggressive incidents:*

The percentage of cases with aggressive incidents varied between hospitals from 3.9% to 8.8% in 2019 and from 4.1% to 10.1% in 2020. The mean SOAS-R score varied between hospitals from 10.0 to 14.1 in 2019 and from 10.2 to 13.7 in 2020. The percentage of aggressive incidents with injury consequences varied between hospitals from 14.3% to 50.3% in 2019 and from 12.7% to 44.1% in 2020.

*In the discussion section, we added the following sentence:*

Another limitation is the very likely presence of unknown confounders. In a previous study on differences between hospitals in the rates of coercive measures, only 27% of the variation could be explained by a wide range of structural data of hospitals and supply areas [24].

Table 2 – some of the p-values appear to be missing.

*Thank you for this hint. We now provide RR and p-values.*

A stronger analysis plan would help strengthen the study’s overall impact and interpretation of findings.

*Thank you for this advice. We extended the analyses section as follows:*

To assess the impact of the COVID-19 pandemic, we analyzed changes in the number of treated cases, involuntary cases, seclusion, restraint, coercive medication, and aggressive incidents between 2019 and 2020. Therefore we …compared the percentage of affected cases and the median (inter-quartile range*,* IQR) duration of coercive measures and the cumulative duration of coercive measures per affected case for 2019 with the respective data for the year 2020.

Manuscript would be strengthened by having an overall conclusion paragraph rather than focusing on limitations.

*Thank you for the suggestion. We added the following paragraph, without shortening the limitations:*

**Conclusions**

The COVID-19 pandemic had detrimental effects on the achievements to avoid coercion during in-patient psychiatric treatment. Necessarily, the focus of attention and rules as well shifted from prevention of coercion to prevention of infections. By and large, this was not due to the admission of patients with COVID-19 or patients with COVID-19-related disorders, but due to the many adverse circumstances of the pandemic for the conditions of humane and respectful in-patient treatment.

Reviewer #3: Thank you for asking me to review this paper.The topic area is important relevant and interesting, the data registry upon which it is based a possible strength. My main observation of this work however is that the statistical analyses as they’re currently presented seem to indicate that the analysis was mainly descriptive, i.e. not taking approaches that could account for confounding (relative risks are mentioned but it does not appear that any regression approaches were used in the analysis). This is a major limitation and at the very least needs to be reflected upon as a concern in the discussion, however it would be preferable if the analytic methods could go further to address this in the manuscript.

Some other comments -
1. Abstract- some context on country would be helpful for an international readership. Perhaps add “a region in Germany” after ‘State of Baden-Wuerttemberg’

*The first paragraph of the introduction explains the country’s conditions related to the topic of coercion. In the second paragraph, we added the following sentence:*

This registry is unique in Germany, as it contains raw data on each coercive measure in all psychiatric hospitals on any legal basis (mental health law, guardianship law, temporary detention).

*In the study design paragraph, we added the following sentence:*

Baden-Wuerttemberg with 11 million inhabitants covers the South West of Germany, with Stuttgart as the State’s capital.

2. The last statement in the abstract ‘The focus of attention has shifted from prevention of coercion to prevention of infection.’ is a little unclear and goes further than what the results suggest

*We would like to keep this statement because it meets the core of our study’s message. We hope that this is better reflected by the newly added conclusion paragraph as suggested by reviewer#2. We amended the sentence in the abstract as follows:*

The focus of attention and internal rules as well have shifted from prevention of coercion to prevention of infection.

Manuscript in general
1. Some typos - manuscript should be proofed carefully for these eg. ‘leaves’ should be leave pg4, under ‘ Study design“ missing the word ‘from’ , etc. Please re-read and check throughout for small grammatical and spelling errors.

*We tried to do so. We had done external professional proofreading before submitting the ms. Unfortunately, it turns out repetitively that we are better in finding others’ mistakes than our own.*

Methods
1. More detail would be helpful- does the registry represent all hospitals in the region , if not which are not represented (proportions?). In Germany would all secondary mental healthcare be provided by these institutions or can people receive secondary care from outside of these institutions?

*We added explanations, see amended text as presented to the suggestion by reviewer #1.*

*Regarding secondary healthcare, we realized that it is necessary to add some explanations on the German healthcare system, particularly in a general journal. We added the following sentences in the introduction, not going too much into the (complex) details of secondary mental healthcare that were not objective of this study:*

In Germany, psychiatric clinics are widely available on a high quality level. About half of these clinics are part of university hospitals or general hospitals, the other half are specialized psychiatric hospitals. Day clinics are widely available, standing alone or as a part of these hospitals. Outpatient treatment is provided by the hospitals for people with severe mental disorders together with community psychiatric services, treatment for milder mental disorders is provided by physicians or psychologists in own practice, funded by health insurances. Community treatment orders or other types of involuntary outpatient commitment are not legalized, so that any type of coercion in association with treatment of mental disorders can only occur within a hospital.

2. Some mention is made of “Staff Observation Aggression Scale – Revised (SOAS-R) “. Although some details are provided with references further detail would be helpful, rather than expecting readers to have to look up the other papers.It would be helpful to know which domains are covered in this scale, who fills out the scale and relevant psychometric properties of the scale. Mean scores are presented later but these are meaningless to readers unfamiliar with this scale.

*Reviewer #2 suggested the same. Please see there the extended text.*

3. This line needs further explanation “Due to data privacy rules, the exact date of incidents was not available so that we could not restrict our analysis to the months of the pandemic (beginning in March, 2020). This may have led to a systematic underestimation of observed changes of about 15%.” If you could not restrict analysis to the month of the pandemic how are you able to determine which events occurred prior to the pandemic (2019) versus During the pandemic (2020)? How did you determine an underestimation percentage of 15%? Please explain this in more detail.

*We slightly extended the explanation to clarify that:*

Data are available for the respective years, but, due to data privacy rules, the exact date of incidents is not provided so that we could not restrict our analysis to the months of the pandemic (beginning in March, 2020). This may have led to a systematic underestimation of observed changes of about 15%.

*It means that we could exactly determine the year, but not the month. Since the pandemic begun about March 2020 (not to be exactly determined), resulting changes came into action only for about 10 out of 12 months of the year (without January and February, accounting for 1/6 (about 15 %) of the time of the year). We think that the explanation should be sufficiently clear in the text, but inserted a further sentence referring to this circumstance in the discussion.*

4. Statistical methods- more detail needed- what package did you use (spss/ stata etc)? How were the relative risks calculated? Presumably these are crude or unadjusted? If these relative risks were calculated through regression models Did you consider adjusting for confounders? Also did you check underlying assumptions for using Cohen’s D were not violated - cohens D has similar assumptions to T tests

*We only calculated crude RR without estimation through regression models. We clarified this in the method section.*

For the differences in the proportions of cases with coercive measures, we calculated unadjusted risk ratios (RR) and 95% confidence intervals (95%-CI), and for the differences in the median cumulated duration of coercive measures and for the difference in the SOAS-R score, we calculated effect sizes eta squared and converted them into common language effect sizes Cohen’s d [17,18]. Analyses were done with IBM® SPSS® Statistics Version 27, Mocrosoft® Excel® 2013. Effect sizes for duration of coercive measures and for the difference in the SOAS-R score were calculated online [18].

5. Tables: rather than RR with P values It would be preferable to have 95% confidence intervals for the RR. This would allow us to see the strength of Association alongside precision.

*Thank you for this advice. We changed the ms. accordingly.*

6. Table 2- first few rows - it is difficult to assess if these absolute differences are meaningful without a denominator

*We now present RRs.*

7. In the tables which statistical tests do the p values relate to? A footnote would be helpful . Rather than showing p=0.000 -standard practice would be to show a boundary eg p<0.001 etc or to two decimal places.

*We changed this.*

8. Discussion - this detail “Now we can present such data based on a total survey of all coercive interventions in psychiatric hospitals in a Federal State with 11 million inhabitants, encompassing over 200,000 cases in the years 2019 and 2020.” Or something equivalent should be presented earlier on eg in methods.

*Thank you for this hint. We extended the method section as follows:*

Hence, this is a full survey, comprising each coercive measure that had been performed in one of the State’s psychiatric hospitals in the respective year in adults. Baden-Wuerttemberg with 11 million inhabitants covers the South West of Germany, with Stuttgart as the State’s capital.

9. The discussion section could State more about the limitations of the study. There are several obvious ones such as confounding which should be mentioned, in addition there are others which the authors could specify

*We added two points to the discussion:*

Another limitation is the very likely presence of unknown confounders. In a previous study on differences between hospitals in the rates of coercive measures, only 27% of the variation could be explained by a wide range of structural data of hospitals and supply areas [22]. With respect to data quality, no findings are available on the reliability of the data collected. It may also be that, despite detailed instructions, there is sufficiently uniform recording across clinics. So underreporting or incorrect reporting cannot be ruled out.

10. Follow STROBE guidelines in your reporting
